# Metastases to the Stomach: Clinicopathologic Features of Metastases Mimicking Gastric Primaries

**DOI:** 10.5146/tjpath.2021.01529

**Published:** 2021-09-15

**Authors:** Ali Yılmaz Altay, Melek Büyük, İlker Özgür, Ali Fuat Kaan Gök, Bilger Çavuş, Esra Aydın, Sezai Vatansever, Mine Güllüoğlu

**Affiliations:** Department of Pathology, Istanbul Faculty of Medicine, Istanbul University, Itanbul, Turkey; Department of Pathology, Istanbul Faculty of Medicine, Istanbul University, Istanbul, Turkey; Department of General Surgery, Istanbul Faculty of Medicine, Istanbul University, Istanbul, Turkey; Department of Internal Medicine (Gastroenterology), Istanbul Faculty of Medicine, Istanbul University, Istanbul, Turkey; Institute of Oncology, Istanbul University, Istanbul, Turkey

**Keywords:** Metastasis, Stomach, Metastatic carcinoma, Endoscopy, Gastric mass

## Abstract

***Objective:*** Metastatic involvement of the stomach is a rare event. Our aim in this study was to document the clinicopathological findings in patients with gastric metastases and find out if there are any potentially significant features to be used in the differential diagnosis.

***Material and Method:*** Our cohort consisted of 17 histologically verified gastric metastasis cases. Clinical, endoscopic and microscopic features were retrospectively analyzed.

***Results:*** The primary sites were the breast, skin, lungs, ovaries, colon, and gluteal soft tissue. Three patients were symptomatic because of the metastatic involvement of the stomach and 9 patients had concomitant metastasis in other sites. Invasive lobular breast carcinoma and malignant melanoma were the most common metastatic malignancies. The most common macroscopic appearance was the diffuse infiltrative type (Borrmann Type 4). Most of the metastatic lesions endoscopically mimicked primary gastric cancer. Furthermore, some of the metastatic lesions, particularly invasive lobular carcinoma of the breast and malignant melanoma, displayed histopathologic features similar to the primary gastric malignancies to a certain extent.

***Conclusion:*** The possibility of metastatic involvement of stomach must be kept in mind while dealing with a gastric mass lesion in a cancer patient, even though the clinical and endoscopic features suggest primary gastric cancer. Our study points out the importance of conveying the information about medical history and clinical findings of the patients for correct pathologic differential diagnosis.

## INTRODUCTION

Metastatic disease involving the stomach is rare. The incidence is less than 5.4% based on the autopsy series of cancer patients (1–5). The cancers that are most commonly associated with gastric metastasis are malignant melanoma, lung adenocarcinoma, and invasive lobular carcinoma of the breast (2,3,6).

Gastric metastases are associated with advanced stage and poor prognosis; however, the low incidence of this event makes management decisions difficult. The same reason also harbors the risk of misdiagnosis in routine pathology practice.

In our study, we reviewed archive materials of gastric biopsies as well as the clinical and endoscopic findings of gastric metastasis patients. Our aim was to create awareness for this rare event in pathology practice and highlight the entities that need to be taken into consideration for the differential diagnosis.

## MATERIALS and METHOD

Our cohort consists of seventeen patients with histologically verified metastatic cancer in the stomach seen at the Istanbul University, Istanbul Faculty of Medicine Hospital between January 2007 and December 2017. Secondary hematolymphoid malignancies were excluded. Pathology reports and archive slides were reviewed. Clinical data of the patients were obtained from the medical records. The national database was used for the survival data. This is a retrospective archive study and does not include any study on live human or animal subjects by any of the authors. Therefore ethics committee approval was not sought.

## RESULTS

### Demography and Tumor Types

There were 12 female and 5 male patients in our cohort. The mean age was 57.41±12.99 years. The most common malignancy metastatic to the stomach was invasive lobular carcinoma of the breast (6/17), followed by malignant melanoma (5/17). The rest of the cohort consisted of lung carcinomas (2/17) (1 adenocarcinoma, 1 squamous cell carcinoma (SCC)), serous ovarian carcinomas (2/17), colorectal adenocarcinoma (1/17) and undifferentiated high-grade soft tissue sarcoma (1/17) (Table 1).

**Table 1:** Clinicopathologic features of gastric metastasis cases.

**Mean Age: 57.41 ±12.99**			**Malignant Melanoma**	**Invasive Lobular Carcinoma**	**Adenocarcinoma**			**Squamous Cell Carcinoma**	**Undifferentiated Pleomorphic Sarcoma**	**Data Unavailable**	**Total** **n=17**
					**Lung**	**Colorectal**	**Ovarian**				
Male			3		1			1		-	17
Female			2	6		1	2		1		
Location within the stomach	Cardia							1		3	14
	Fundus					1					
	Corpus		3	2	1				1		
	Antrum		1	3			1				
Solitary/Multiple	Solitary		2	5	1	1	1	1	1	3	14
	Multiple		2								
Synchronous metastasis	Yes		4	3		1			1	5	12
	No				1		1	1			
Endoscopic appearance	Submucosal mass like appearance									3	14
	Primary gastric carcinoma-like appearance	Borrmann Type 1	1			1		1	1		
		Borrmann Type 2	2	1							
		Borrmann Type 3	1				1				
		Borrmann Type 4		4	1						

### Clinical Findings

In our cohort, only three patients were symptomatic related to the metastatic lesion and required surgical intervention. One of the metastatic malignant melanoma patients presented with upper gastrointestinal bleeding. One of the invasive lobular carcinoma metastases caused gastric outflow obstruction that needed an emergency gastrectomy and the colonic adenocarcinoma metastatic to the stomach caused fistula formation between the pleural cavity and the stomach.

Invasive lobular carcinoma and malignant melanoma were the two most common malignancies with synchronous metastasis in our cohort. Synchronous metastatic sites for malignant melanoma were the axillary lymph nodes and bowel; for invasive lobular carcinoma they were bone and bowel; for colonic adenocarcinoma it was the liver, and for soft tissue sarcoma it was the heart. Both high-grade serous ovarian adenocarcinoma cases had peritoneal involvement. Peritoneal involvement was not present in the other malignancies in our cohort.

Information about the time period between the diagnosis of the primary tumor and gastric metastasis was available for 9 cases (1 malignant melanoma, 3 invasive lobular carcinomas, 1 SCC, 1 lung adenocarcinoma, 2 high-grade serous adenocarcinomas and 1 soft tissue sarcoma). The longest period was 60 months for two invasive lobular carcinoma and SCC cases. The shortest period was 7 months for malignant melanoma. For the other cases, the values were 36 months for high-grade serous adenocarcinomas, 24 months for lung adenocarcinoma and one invasive lobular carcinoma, and 12 months for soft tissue sarcoma.

### Endoscopic Findings

**Figure 1:**
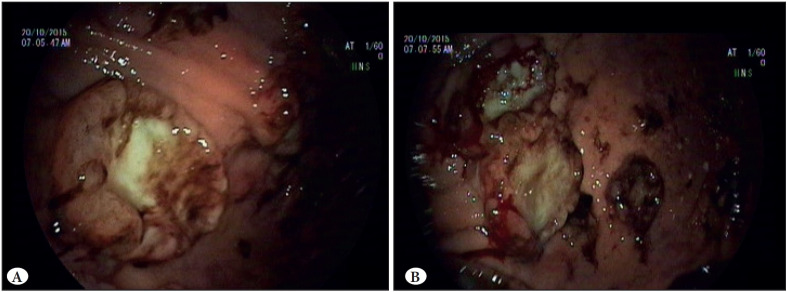
A,B) Endoscopic appearance of malignant melanoma metastasis in the gastric antrum. Multiple metastatic lesions displaying heterogenous pigmentation.

The endoscopic findings of 14 patients out of 17 were obtained from the medical records. Two melanoma cases had multiple stomach lesions (Figure 1) whereas the lesions in the other cases were solitary at the time of the diagnosis.

**Figure 2:**
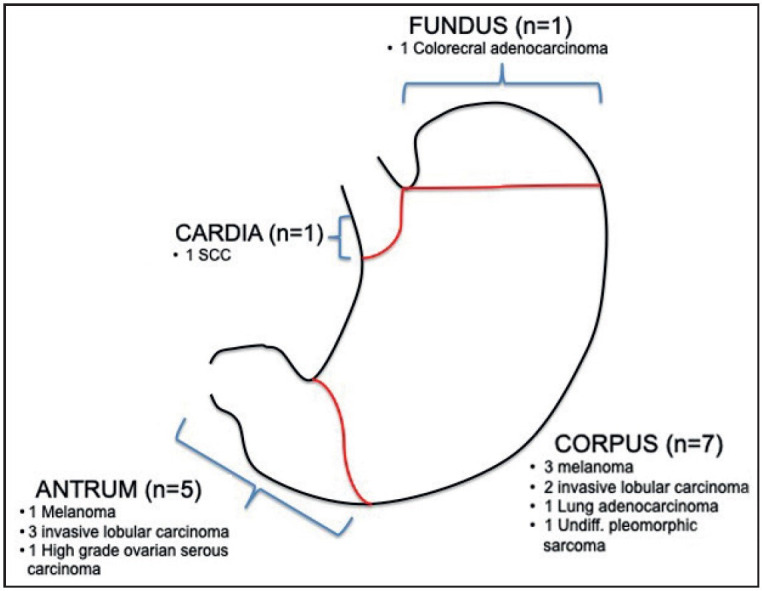
Anatomic distribution of gastric metastases

As we evaluated the distribution of the lesions, the lung SCC metastasis was located at the cardia; the colorectal carcinoma metastasis was located at the fundus; metastases of three melanomas, two invasive lobular carcinomas, one lung adenocarcinoma and sarcoma were located at the corpus and those of one melanoma, one serous ovarian carcinoma, and three invasive lobular carcinomas were located at the antrum (Table 1) (Figure 2).

**Figure 3:**
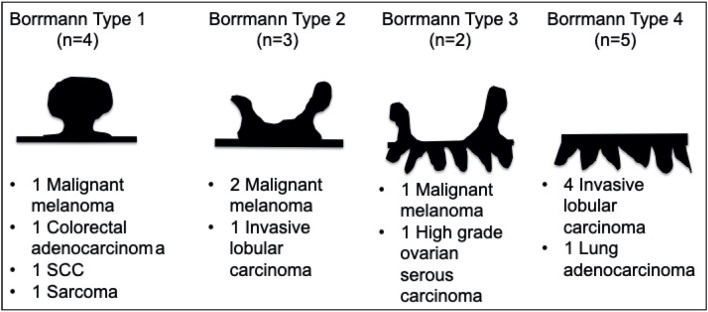
Macroscopic appearance of gastric metastases according to the Borrmann Classification

We detected that the endoscopic features resembled primary gastric carcinoma in all cases. Four cases (28%) (one melanoma, one colorectal carcinoma, one lung SCC, and one sarcoma) presented as polypoid masses that fit into the Borrmann type 1 category. Three (21%) of the lesions (2 melanomas and 1 invasive lobular carcinoma) were well-defined ulcerative lesions that fit into the Borrmann type 2 category. Two (14%) of the lesions (1 melanoma and 1 serous ovarian carcinoma) were ulcerative lesions with infiltrating margins that fit into the Borrmann type 3 category. Five cases (35%) (4 invasive lobular carcinomas and 1 lung adenocarcinoma) had a diffuse infiltrating appearance that fit into the Borrmann type 4 category (Table 1) (Figure 3).

### Histopathological Findings

**Figure 4:**
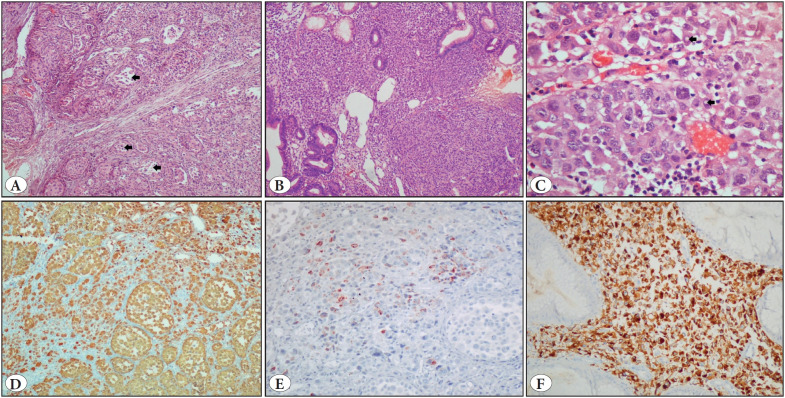
Malignant melanoma metastasis. **A)** Melanoma cells forming nests of varying sizes. Some nests display pseudoglandular pattern (arrows) (H&E; x100). **B)** Neoplastic cells infiltrating the lamina propria. Neoplastic cells are small and show minimal pleomorphism (H&E; x100). **C)** Melanoma cells showing prominent nuclear pleomorphism and intranuclear inclusions (arrows) (H&E; x400). **D)** Neoplastic cells displaying diffuse S100 protein immunoreactivity (IHC; x100). **E)** Focal cytoplasmic HMB-45 immunoreactivity (IHC; x100). **F)** Diffuse cytoplasmic Melan A immunoreactivity (IHC; x200).

Among the malignant melanoma cases, only one displayed prominent melanin pigment in neoplastic cells. The remaining four melanoma cases appeared as amelanotic. Four cases showed diffuse/interstitial growth pattern. Nested and pseudoglandular appearance was observed in only one. Nuclear pleomorphism was detected in all cases. Bizarre cells and rhabdoid morphology were observed in one case, prominent nucleoli in four, and intranuclear pseudoinclusions in two cases. All cases were positive for S100 protein, melan-A and HMB-45 (Figure 4).

Metastases of epithelial malignancies displayed morphologic and immunohistochemical features similar to their primary tumors.

**Figure 5:**
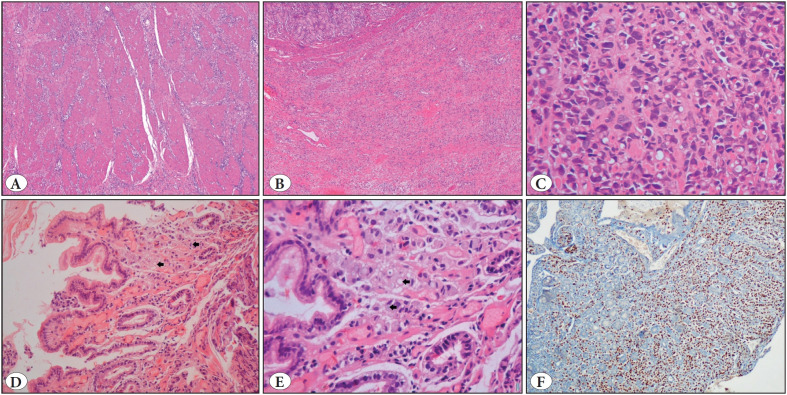
Invasive lobular carcinoma metastasis to the stomach. **A)** Diffuse infiltration of neoplastic cells within the muscle layer (H&E; x40). **B)** Submucosal infiltration of neoplastic cells (H&E; x40). **C)** Neoplastic cells with intracytoplasmic lumina containing eosinophilic globular material (H&E; x400). **D)** Neoplastic cells display subtle nuclear atypia forming strands in the lamina propria (arrows) (H&E; x200). **E)** The same neoplastic cells seen in Figure 1, mimicking signet ring cells with intracytoplasmic mucoid material and lumen formation (arrows) (H&E; x400). **F)** GATA3 nuclear immunopositivity in neoplastic cells (IHC; x200).

All invasive lobular carcinoma metastases displayed similar morphologic features (i.e. tumor cells appearing as single cells or forming cords). Intracytoplasmic lumen formation was detected in all cases. Three cases had prominent eosinophilic secretory material in the cytoplasm of tumor cells and other three cases had mucoid PAS positive material within the intracytoplasmic lumina (Figure 5).

Squamous cell carcinoma metastasis showed no keratinization and displayed poorly differentiated areas.

The lung adenocarcinoma metastasis was composed of solid groups of tumor cells without any glandular formations and displayed cytokeratin 7 and thyroid transcription factor 1 (TTF-1) immunopositivity.

The colorectal adenocarcinoma metastasis showed the usual colorectal adenocarcinoma morphology with glands composed of columnar cells and necrotic debris in the glandular lumina. Tumor cells were cytokeratin 7 negative but cytokeratin 20, CDX2, and carcinoembryonic antigen (CEA) positive.

Both ovarian serous carcinoma cases were of the high-grade type.

In the metastatic sarcoma case, the primary tumor that had been in the gluteal region was composed of pleomorphic spindle cells devoid of any particular histologic pattern, and thorough immunohistochemical evaluation showed no particular line of differentiation. Its gastric metastasis had spindle cells in a myxoid background and was more hypocellular than its primary.

## DISCUSSION

The stomach is an unusual site for metastasis. The incidence rate of metastatic stomach masses is 0.2-0.8% (3–5). However, the exact incidence is unknown, as the stomach is not routinely evaluated for metastatic cancer. Patients with gastric metastasis are usually asymptomatic (7). Symptomatic patients usually have stomach pain, nausea, vomiting, bleeding, and symptoms related to obstruction (2).

As we stated previously, gastric metastasis is a late event and associated with an advanced stage (1). In our cohort, 9 patients had biopsy-proven metastases other than in the stomach. Although we do not have the data for all cases, the time period between the first diagnosis of the primary tumor and the gastric metastasis was longest for SCC and invasive lobular carcinoma cases and shortest for malignant melanoma cases. This feature can be attributed to the overall aggressiveness of the mentioned neoplasm.

We encountered solitary gastric metastatic lesions more commonly than multiple lesions. This finding correlates with another study in the literature (2). Macroscopic appearance of the lesions was similar to the primary gastric tumors. Lobular carcinoma metastasis in particular seems to have a tendency to create the linitis plastica appearance. Four out of 6 lobular carcinoma metastases displayed an endoscopic appearance mimicking the diffuse infiltrative type of primary gastric carcinoma. This feature was shared with the lung adenocarcinoma metastasis as well. Other metastatic masses showed various endoscopic appearances and a polypoid mass lesion seemed the most common one. Although the exact relationship between tumor biology and macroscopic appearance of metastatic masses is not fully understood, the discohesive nature of the lobular carcinoma cells can be pointed out as the reason for diffuse infiltrative growth pattern in metastatic site (6). As the metastatic masses are more commonly presented as solitary mass lesions that resemble any of the Borrmann types of primary gastric adenocarcinoma, it is sometimes difficult to differentiate a metastatic gastric mass from a primary gastric malignancy endoscopically. The endoscopist, as well as the pathologist, should be well aware of the possibility of metastases to the stomach - although rare - while dealing with a cancer patient with a gastric mass.

Theoretically, any tumor might metastasize to the stomach. Malignant melanoma, invasive lobular breast carcinoma and lung carcinomas are the most commonly reported primaries as stated before (2,3,6). However, gastric metastatic tumors originating from the kidney, pancreas, esophagus, skin, testis, cervix, and colon have also been reported (1,2,8).

Invasive lobular carcinoma deserve special attention because of both the macroscopic and microscopic pathological findings, which are sometimes indistinguishable from those of primary gastric carcinoma (9–13). The clinical history plays an important role in differentiating between primary and metastatic masses. However, there are cases that the metastatic lesion was diagnosed before the primary (14). An immunohistochemical panel consisting of estrogen receptor (ER), progesterone receptor (PR), gross cystic disease fluid protein (GCDFP-15), mammoglobin, and GATA3 can be useful in differentiating invasive lobular breast carcinoma (15–19). However, one should be well aware of the fact that weak ER positivity does not rule out gastric adenocarcinoma (20,21). Yokozaki et al. detected that immunexpression of ER was a more frequent finding in poorly differentiated gastric adenocarcinoma than that of its well differentiated counterpart and immunoreactivity was not sex-related (21). However, ER was found to be negative in all gastric adenocarcinomas in a European study (22). Because of the morphologic similarities between primary diffuse type gastric carcinoma and invasive lobular carcinoma of the breast, the possibility of a metastatic breast carcinoma - although low - must be kept in mind for the differential diagnosis.

Malignant melanoma has diverse morphologic appearances. We found that the tumor cells in all melanoma cases had marked nuclear pleomorphism and prominent nucleoli. Clinical history plays an important role in diagnosing metastatic malignant melanoma in the stomach but the possibility of an occult melanoma or total regression of primary lesion must be kept in mind. Melanomas can be separated from gastric carcinomas by their S100 protein expression staining coupled with their lack of keratin immunoreactivity. More specific melanoma markers such as Melan-A, HMB-45, SRY-related HMG-box 10 (SOX-10), microphthalmia-associated transcription factor (MiTF) and tyrosinase, should be used together with S100 protein for accurate differential diagnosis. As melanomas can also be immunoreactive to c-kit/CD117 antibodies, a panel approach is warranted to exclude epitheloid gastrointestinal stromal tumor (GIST). Melan-A can be positive on GISTs as well (23).

About 31-38% of ovarian cancer patients develop distant metastasis during the course of the disease (24,25). The most common ovarian adenocarcinoma subtype metastatic to the stomach is serous adenocarcinoma (26). Because of its tendency to spread through peritoneal surfaces, gastric metastasis of ovarian serous adenocarcinoma without peritoneal involvement is extremely rare (24–26). Both of our metastatic ovarian carcinoma cases were serous adenocarcinomas and had gastric peritoneal surface involvement.

There are occasional reports of primary gastric undifferentiated sarcomas (27). However, metastatic sarcomas in the stomach are extremely rare (28–30). In our literature review, we found 28 cases of sarcoma metastasis to stomach between 1983 and 2016 in the Pubmed database (Table 2). We revealed that the primary site was the lower extremity in 11 out of 28 cases; however, the location of the primary had no impact on the prediction of gastric metastatic potential when all 28 metastatic sarcoma cases were taken into account. The primary lesion was located at the right gluteal area in our only metastatic sarcoma case. When we look at the distribution of the metastatic lesions in our literature review, 12 of the metastatic lesions were located at the curvatures of the stomach (1 lesser and 11 greater). Akatsu et al. suggests that this distribution seems to reflect the tendency of hematogenous spread of sarcomas occurs through the gastroduodenal or gastroepiploic vessels (31). The metastatic gastric lesion in our sarcoma case was located in the lesser curvature at the cardia-corpus junction.

**Table 2:** Literature review of metastatic sarcoma cases.

**Authors**	**Age**	**Sex**	**Histologic Subtype**	**Primary Site**	**Duration (Months)**	**Site within stomach**
Akatsu et al. (2006) (31)	75	Male	MFH	Back	24	Antrum
Dent et al. (2010) (32)	60	Male	Undifferentiated pleomorphic sarcoma	Left posterior shoulder	6	Greater curvature
Overberg-Schmidt et al. (1999) (33)	11	Female	Osteosarcoma	Femur	14	Posterior gastric wall
Strong et al. (2007) (34)	17	Male	Osteosarcoma	Femur	30	Antrum
Horiuchi et al. (2010) (35)	18	Male	Osteosarcoma	Humerus	30	Corpus
Urakawa et al. (2013) (36)	73	Male	Osteosarcoma	Sternum	11	Corpus
Moses et al. (2013) (37)	17	Male	Osteosarcoma	Leg	14	Fundus
Abe et al. (2016) (38)	78	Female	Leiomyosarcoma	Heart, left atrium	24	-
Costa et al. (2016) (39)	52	Female	Leiomyosarcoma	Uterus	84	Corpus, greater curvature
Nakajima et al. (2005) (40)	61	Male	Leiomyosarcoma	Leg	24	Corpus
Ferrozi et al. (1994) (41)	50	Female	Leiomyosarcoma	Retroperitoneum	6	Greater curvature
Ferrozi et al. (1994) (41)	55	Male	Leiomyosarcoma	Retroperitoneum	24	Antrum, greater curvature
Obata et al. (1993) (42)	58	Female	Angiosarcoma	Left hip	2	Corpus, greater curvature
Kim et al. (2005) (43)	54	Male	Angiosarcoma	Liver	3	Antrum, cardia
Okabayashi et al. (1993) (44)	84	Male	Angiosarcoma	Thoracic wall	11	Antrum
Konishi et al. (1994) (45)	34	Female	Chondrosarcoma	Popliteal fossa	84	Immediately below GEJ
Chuan et al. (2016) (46)	56	Male	Synovial sarcoma	Thigh	12	Incisura angularis
Choi et al. (2016) (47)	44	Female	Phylloides tumor	Breast	60	Lesser curvature
Sonobe et al. (1980) (48)	39	Male	MFH	Maxillary sinus	12	Corpus
Adams et al. (1983) (49)	70	Female	MFH	Upper thigh	36	Greater curvature
Yasuda et al. (1985) (50)	66	Male	MFH	Lung	-	Body, greater curvature
Fujii et al. (1993) (51)	77	Male	MFH	Lung	-	Antrum
Okada et al. (1995) (52)	67	Female	MFH	Femur	-	Body, greater curvature
Ohuchi et al. (1997) (53)	62	Male	MFH	Ethmoid sinus	-	Body, antrum, greater curvature
Hisai et al. (1998) (54)	64	Male	MFH	Knee	-	Antrum, greater curvature
Nishida et al. (1998) (55)	69	Male	MFH	Mesentery	-	-
Kato et al. (2002) (56)	70	Male	MFH	Gallbladder	29	Antrum, greater curvature
Ibanez et al. (2011) (57)	39	Male	MFH	Thigh	60	Antrum, corpus

**MFH:** Malignant fibrous histiocytoma, GEJ: Gastroesophageal junction

As we stated previously, information about the clinical history of the patient is crucial in the differential diagnosis. None of the morphologic features in our cohort was pathognomonic except those of the pigmented melanoma case. All other cases can easily be misdiagnosed as poorly differentiated adenocarcinoma without the clinical information. This problem becomes more apparent for invasive lobular breast carcinoma cases because of their almost identical morphologic features with poorly cohesive gastric carcinoma.

In this study we highlighted the similarities of primary gastric cancer and metastatic malignancies to the stomach in endoscopic biopsies. There are other studies with similar or larger cohorts, including autopsy series, in the literature (2,8,58,59). These studies mainly focus on epidemiological, endoscopic and macroscopic aspects of the metastatic lesions of the stomach without going into the details of the microscopic features of the cases. In this study, we analyzed the histologic features of the metastases and potential microscopic pitfalls in the differential diagnosis as well as the endoscopic and macroscopic findings.

Despite its low prevalence, the possibility of gastric metastasis must be kept in mind for gastric masses with unusual macroscopic and microscopic features. Our study also points out the importance of the clinical history and clinical information in the decision-making process of pathological differential diagnosis.

## Conflict of INTEREST

The authors declare no conflict of interest.

## FUNDING

None
